# High leaf mass per area *Oryza* genotypes invest more leaf mass to cell wall and show a low mesophyll conductance

**DOI:** 10.1093/aobpla/plaa028

**Published:** 2020-06-19

**Authors:** Miao Ye, Zhengcan Zhang, Guanjun Huang, Zhuang Xiong, Shaobing Peng, Yong Li

**Affiliations:** 1 Ministry of Agriculture Key Laboratory of Crop Ecophysiology and Farming System in the Middle Reaches of the Yangtze River, College of Plant Science and Technology, Huazhong Agricultural University, Wuhan, Hubei, China; 2 National Key Laboratory of Crop Genetic Improvement, Huazhong Agricultural University, Wuhan, Hubei, China

**Keywords:** Cell wall, leaf anatomy, leaf chemical compositions, leaf mass per area, *Oryza sativa* L, tradeoff

## Abstract

The intraspecific variations of leaf structure and anatomy in rice leaves and their impacts on gas diffusion are still unknown. Researches about the tradeoff between structural compositions and intracellular chemical components within rice leaves are still lacking. The objectives of the present study were to investigate the varietal differences in leaf structure and leaf chemical compositions, and the tradeoff between leaf structural tissues and intracellular chemical components in rice leaves. Leaf structure, leaf anatomy, leaf chemical composition concentrations and gas exchange parameters were measured on eight *Oryza sativa* L. genotypes to investigate the intraspecific variations in leaf structure and leaf anatomy and their impacts on gas exchange parameters, and to study the tradeoff between leaf structural compositions (cell wall compounds) and intracellular chemical components (non-structural carbohydrates, nitrogen, chlorophyll). Leaf thickness increased with leaf mass per area (LMA), while leaf density did not correlate with LMA. Mesophyll cell surface area exposed to intercellular airspace (IAS) per leaf area, the surface area of chloroplasts exposed to IAS and cell wall thickness increased with LMA. Cell wall compounds accounted for 71.5 % of leaf dry mass, while mass-based nitrogen and chlorophyll concentrations decreased with LMA. Mesophyll conductance was negatively correlated with LMA and cell wall thickness. High LMA rice genotypes invest more leaf mass to cell wall and possess a low mesophyll conductance.

## Teaser

This study reports evidence that high LMA rice plants invest more leaf mass to cell wall and possess a low mesophyll conductance. Leaf thickness rather than leaf density is the main driving factor for difference in LMA in the present study. The surface areas of mesophyll cells and chloroplasts facing intercellular airspaces and cell wall thickness all increase with leaf thickness. Thick cell wall leads to a low mesophyll conductance and mass-based photosynthetic rate. Cell wall compounds account for the majority of the leaf dry mass in rice leaves.

## Introduction

Under current ambient conditions (around 400 μmol CO_2_ mol^-1^ air), CO_2_ diffusion conductance from the air to the sites of carboxylation is regarded as one of the most important limiting factors for photosynthesis in C_3_ plants (Evans *et al.* 2009; Li *et al.* 2009; Yamori *et al.* 2011; Flexas *et al.* 2012; Adachi *et al.* 2013).

After reaching substomatal cavities, CO_2_ needs to further diffuse through the mesophyll cell to reach the carboxylation sites (Terashima *et al.* 2011). The CO_2_ diffusion resistance from the substomatal cavities to the carboxylation sites is called mesophyll resistance (*r*_m_), and the reciprocal of *r*_m_ is mesophyll conductance (*g*_m_). *g*_m_ is determined by both anatomical and biochemical components (e.g. aquaporins and carbonic anhydrase etc.) (Nakhoul *et al.* 1998; Uehlein 2003, Evans *et al.* 2009). There are many anatomical properties relating to mesophyll conductance, including the fraction of intercellular airspace (*f*_ias_), the mesophyll cell wall surface area exposed to intercellular airspace per leaf area (*S*_m_), the surface area of chloroplasts exposed to intercellular airspace (*S*_c_) and cell wall thickness (Evans *et al.* 2009; Scafaro *et al.* 2011; Terashima *et al.* 2011; Peguero-Pina *et al.* 2012; Tosens *et al.* 2012; Muir *et al.* 2014). Cell wall thickness is particularly important as cell wall resistance accounts for about half of the total mesophyll resistance (Terashima *et al.* 2011). Studies also showed that leaves with thicker cell wall usually have higher leaf mass per area (LMA; Terashima *et al.* 2006; Tosens *et al.* 2012; Onoda *et al.* 2017). LMA is found to be negatively correlated with *g*_m_ in some studies (Hassiotou *et al*. 2009, 2010; Muir *et al.* 2014) which is suggested to be related to cell wall thickness and intercellular airspace (Terashima *et al.* 2006; Tosens *et al.* 2012; Onoda *et al.* 2017). However, no correlation between LMA and *g*_m_ is also found in some studies (Hanba *et al.* 2002; Terashima *et al.* 2006; Tomás *et al.* 2013; Fini *et al.* 2016; Peguero-Pina *et al.* 2017; Ren *et al.* 2019), which may relate to different physiological and biochemical features.

According to its definition, LMA is the product of leaf thickness (LT) with leaf density (LD). Therefore, the variation of LMA can result from variation in LD, in LT or both. By compiling data on 6100 LMA values from 3800 species, Poorter *et al.* (2010) illustrated that LD can explain 80 % and LT explain 20 % of the differences in LMA. For *Oryza sativa*, Xiong *et al.* (2016*b*) showed that LMA are positively related to both LD and LT in 14 genotypes, but the correlation with LD was stronger. However, two nitrogen levels were set in their research, and nitrogen levels also impacted LT, LD and LMA, making the cause for variations in rice LMA still unknown. LT and LD can both impact *g*_m_, for example, Ren *et al.* (2019) found that *g*_m_ was negatively related to LT by analysing data from previous 44 publications. The influences of LT and LD on *g*_m_ are mainly driven by the changes in leaf internal anatomy, including *S*_m_, *S*_c_ and *f*_ias_.

Leaf chemical composition is also an important component of variation in LMA and has been used to explore the tradeoff between structural tissues, mainly including cell wall compounds, and intracellular chemical components, including non-structural carbohydrates and especially some components like nitrogen and chlorophyll which are associated with photosynthesis. For example, high LMA leaves tended to contain more structural tissues while less inclusions (minerals, organic acids, soluble proteins) (Poorter *et al.* 2010). And high LMA species had lower concentrations of cytoplasmic compounds and higher concentrations of cell wall compounds than low-LMA species (Mediavilla *et al.* 2008; Poorter *et al.* 2010). A higher sclerenchymatic tissue fraction (van Arendonk and Poorter 1994), rather than smaller cell sizes in high-LMA species (Castro-Díez *et al.* 2000), was supposed to be the reason. However, relevant studies in rice plants are rare. Therefore, the second objective of the present study is to investigate the tradeoff between structural tissues and intracellular chemical components among rice genotypes.

In the present study, eight rice genotypes with different LMAs were grown in pot experiment to investigate: (i) varietal differences in leaf structure and anatomy and their impacts on mesophyll CO_2_ diffusion and photosynthesis in rice leaves; (ii) leaf chemical compositions in rice leaves; and (iii) the tradeoff between leaf structural tissues and intracellular chemical components in rice leaves.

## Methods

### Plant materials

The experiment was conducted in Huazhong Agricultural University (114.37°E, 30.48°N), Wuhan, Hubei province, China. Eight rice genotypes including Sab Ini, Nucleoryza, Champa, Kirmizi Celtik, Huayou 675, Huanghuazhan, Teqing and Yongyou 12 were used. Rice plants were grown from April to August in 2015. After germination on moist filters, seeds were transferred to nursery plates. When the seedlings had developed an average of three leaves, they were transplanted to 11 L pots with a density of three hills per pot and two seedlings per hill. There were 10 pots per genotype, and each pot filled with 10 kg of soil. Phosphorus (P) and potassium (K) were applied as basal fertilizers at an amount of 1.5 g pot^-1^. N was applied at the amount of 2 g N pot^-1^, 40 % of which was applied as a basal fertilizer and another two topdressings of 30 % each were applied at mid-tillering and the heading stages. Plants were watered daily, and a minimum 2 cm water layer was maintained to avoid drought stress. Pests were intensively controlled using chemical pesticides. Rice plants were grown outdoor and the gas exchange measurements were conducted in a growth chamber (photosynthetic photon flux density (PPFD) 1000 µmol m^-2^ s^-1^ at the leaf level; temperature 28 °C; relative humidity 65 %; CO_2_ concentration 400 µmol mol^-1^) to avoid the influence of changing environment on gas exchange parameters. All measurements were conducted on the newly expanded flag leaves from three different pots of each genotype.

### Gas exchange measurements

A portable photosynthesis system (LI-6400XT, LI-CORInc., Lincoln, NE, USA) with an integrated fluorescence leaf chamber (Li-6400–40; Li-Cor) was used to measure gas exchange and chlorophyll fluorescence on flag leaves between 08:00 and 12:00. Measurements began after the plants had acclimatized to the chamber for approximately 2 h. In the LI-6400XT cuvette, ambient CO_2_ concentration was controlled and set to 400 μmol mol^-1^, leaf temperature was maintained at 28 °C, PPFD was 1500 μ mol m^-2^ s^-1^ and the flow rate was 500 μmol s^-1^. After reaching a steady state, usually takes 25 min, gas exchange parameters, steady-state fluorescence (*F*_s_) and the maximum fluorescence (*F*’ _m_) were recorded with a light saturating pulse of 8000 μmol m^-2^ s^-1^. The actual photochemical efficiency of photosystem II (Φ _PSII_) was calculated as follows:


$$\begin{array}{l} \Phi_{PSII}=\frac{{F^{\prime }}_{m}-F_{s}}{{F^{\prime }}_{m}} \end{array}$$ (1)

The electron transport rate (*J*) was calculated as follows:


$$\begin{array}{l} J=\;PPFD*\alpha \beta *\Phi_{PSII} \end{array}$$ (2)

where α is the leaf absorptance and β is the partitioning of absorbed quanta between photosystem II and photosystem I. The product αβ was determined from the slope of the relationship between Φ _PSII_ and the quantum efficiency of CO_2_ uptake (Φ _CO2_), obtained by varying light intensity under non-photorespiratory conditions at <2 % O_2_ (Valentini *et al.* 1995).

The variable *J* method described in Harley *et al.* (1992) was used to calculate *C*_*c*_ and *g*_m_. *C*_*c*_ and *g*_m_ were calculated as follows:


$$\begin{array}{l} C_{c}=\frac{\Gamma^{*}[J+8(A+R_{d})]}{J-4(A+R_{d})} \end{array}$$ (3)

$$\begin{array}{l} g_{m\;}=\frac{A}{C_{i}-C_{c}} \end{array}$$ (4)

where Γ* represents the CO_2_ compensation point in chloroplasts without day respiration. The day respiration (*R*_d_) and the apparent CO_2_ photocompensation point ($C_{i}^{*}$) were determined using the Laisk method (Brooks and Farquhar 1985). Briefly, *A*/*C*_*i*_ curves were measured over the linear portion of the response curve (at 100, 80, 50 and 25 μmol CO_2_ mol^-1^ air) over three PPFDs (150, 300 and 600 μmol m^-2^ s^-1^) with an LI 6400-02B chamber (Li-Cor), and then linear regressions to the responses for each PPFD were fitted for individual leaves. The intersection point of three *A*/*C*_*i*_ curves was considered as $C_{i}^{*}$ (*x*-axis) and *R*_d_ (*y*-axis) (von Caemmerer *et al.* 1994). Γ* was calculated as follows:


$$\begin{array}{l} \Gamma^{*}=C_{i}^{*}+\frac{R_{d}}{g_{m}} \end{array}$$ (5)

### Leaf anatomy

Three leaves were detached immediately after the gas exchange measurements to determine leaf anatomy, leaf area was measured using a LI-Cor 3000C (LI-COR Inc., Lincoln, NE, USA) leaf area analyzer. Leaves were then oven-dried at 80 °C until they achieved a constant weight (3 days). Afterwards, leaf dry mass was weighed, and LMA was calculated as the ratio of leaf dry mass to leaf area.

Paraffin sections were made for three leaves per genotype to observe leaf anatomy using light microscope (LM). After gas exchange measurements, leaf sections of about 10.0 mm length were cut from the middle of flag leaves and fixed in FAA buffer (38 % formaldehyde, glacial acetic acid and 70 % alcohol) at 4 °C for 24 h, and then were vacuumed in a vacuum chamber (DZF-6050, Shanghai Hasuc Co., Ltd, Shanghai, China). The samples were embedded in paraffins and the leaf cross-sections were made by professionals from Wuhan Google Biotechnology Co. Ltd. The paraffin sections were examined at ×200 magnification with an Olympus IX71 light microscope (Olympus Optical, Tokyo, Japan).

For transmission electron microscope images (TEM), small leaf sections of 4.0 × 1.2 mm were cut from the middle of flag leaves (avoiding midribs) after gas exchange measurements. The leaf sections were infiltrated with fixative 2.5 % (v/v) glutaric aldehyde in 0.1 M phosphate buffer (pH = 7.6) in a vacuum chamber (DZF-6050, Shanghai Hasuc Co., Ltd, Shanghai, China) for 2 h. Ultrathin sections were made by professionals from Core Facility Center and Technical Support, Wuhan Institute of Virology, Chinese Academy of Sciences. Images were acquired using a Tecnai G^2^ 20 TWIN (FEI Co., USA).

LM pictures (×200) were used to measure the area of leaf cross-section and the width of leaf cross-section using ImageJ software (Schneider *et al.* 2012). Three leaves per genotype were measured. LT and LD were calculated as follows:


$$\begin{array}{l} LT=\frac{Area\;of\;leaf\;cross\text{-}section}{Width\;of\;leaf\;cross\text{-}section} \end{array}$$ (6)

$$\begin{array}{l} LD=\frac{LMA}{LT} \end{array}$$ (7)

LM and TEM pictures were used to measure *S*_m_, *S*_c_ and *f*_ias_. The total length of mesophyll cell wall exposed to intercellular airspace (*l*_m_), the total area of intercellular airspace (*S*_ias_), the width of the analysed leaf cross-section (*L*, from the middle of two minor veins to the next one) and the area of the analysed leaf cross-section (*S*) were measured in ×200 LM pictures using ImageJ software. Three leaves per genotype were measured. The fraction of intercellular air space (*f*_ias_) was calculated as:


$$\begin{array}{l} f_{ias}=\frac{S_{ias}}{S} \end{array}$$ (8)

**Fig. 1.**
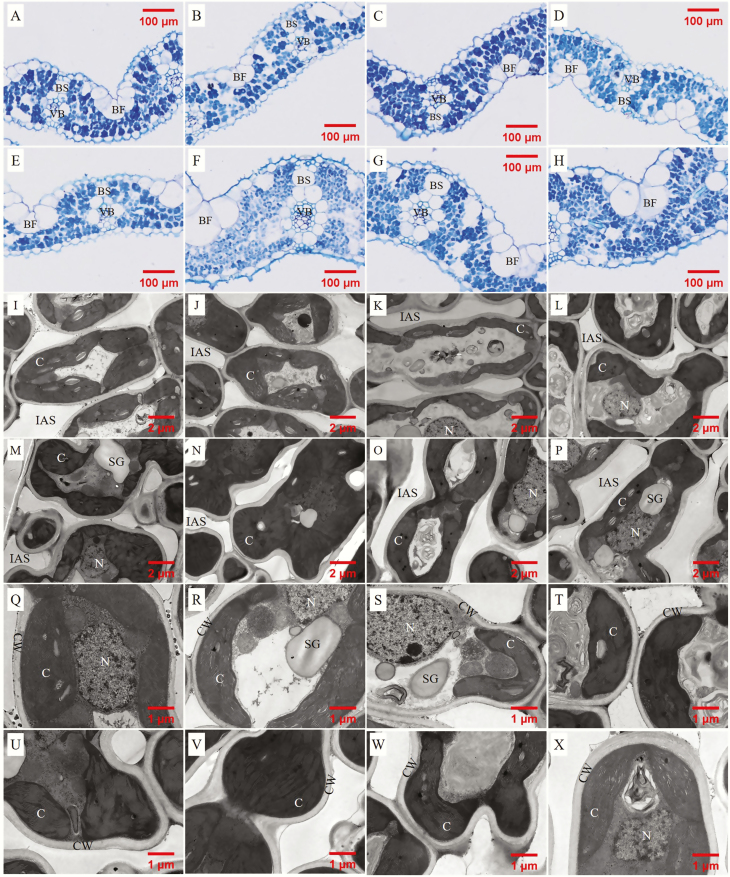
Light (A, B, C, D, E, F, G, H) and transmission electron (1700× (I, J, K, L, M, N, O, P), 3500× (Q, R, S, T, U, V, W, X)) microscope images of leaves detached from Sab Ini, Nucleoryza, Champa, Kirmizi Celtik, Huayou 675, Huanghuazhan, Teqing and Yongyou 12, respectively. IAS, intercellular air space; CW, cell wall; C, chloroplast.


*S*_m_ was calculated as follows:


$$\begin{array}{l} S_{m}=F\times \frac{l_{m}}{L_{}} \end{array}$$ (9)

where *F* is the curvature correction factor, taken to be 1.42 according to previous studies (Scafaro *et al.* 2011; Giuliani *et al.* 2013; Xiong *et al*. 2015, 2016*a*). The proportion of mesophyll cell periphery covered by chloroplasts (*S*_c_/*S*_m_) was analysed in ×1700 TEM pictures (I to P in Fig. 1) using ImageJ software. In total, 5–7 pictures per genotype were analysed. *S*_c_ was calculated as follows:

$$\begin{array}{l} S_{c}=S_{m}\times \frac{S_{c}}{S_{m}} \end{array}$$ (10)

cell wall thickness (CWT) were measured in ×3500 TEM pictures (Q to X in Fig. 1) using ImageJ software. A total of 5–13 pictures (at least 5–13 cells) were measured in each genotype.

### Leaf chemical compositions

Leaves for measuring LMA were used to determine leaf N content. Leaf N content based on leaf mass (N_mass_, %) was measured with an Elementar Vario MAX CN analyzer (Elementar Analysesysteme GmbH, Hanau, Germany), and N_area_ was calculated by multiplying N_mass_ with LMA. Photosynthetic nitrogen use efficiency (PNUE) was calculated as follows:


$$\begin{array}{l} PNUE=\frac{A_{area}}{N_{area}} \end{array}$$ (11)

After gas exchange measurements, the middle part of the flag leaves (0.05–0.1 g) was detached and was put into a 25 mL cuvette to determine the chlorophyll content. About 25 mL 95 % (v/v) ethanol was added into the cuvette. Then the cuvette was covered up and placed in darkness for about 20 h to decolorize the leaves until the background was colorless. Then the extract was transferred to a 50 mL brown volume bottle, the leaves and cuvette were washed with 95 % (v/v) ethanol and the ethanol was poured into the volume bottle, which was finally filled to 50 mL with 95 % (v/v) ethanol. Spectrophotometry was determined with a UV–Vis spectrophotometer (UV-2102C, UNICO., USA) under 665, 649, 470 nm wavelength. All measurements were conducted in dark to avoid chlorophyll’s decomposition. The content of chlorophyll a (*C*_a_), chlorophyll b (*C*_b_) and carotenoid (*C*_x·c_) were calculated as follows:


$$\begin{array}{l} C_{a}=\;13.95A_{665}-6.88A_{649} \end{array}$$ (12)

$$\begin{array}{l} C_{b}=\;24.96A_{649}-7.32A_{665} \end{array}$$ (13)

$$\begin{array}{l} {C_{x}}_{\cdot c}=\frac{1000A_{470}-2.05C_{a}-114.8C_{b}}{245} \end{array}$$ (14)

Contents of soluble sugars and starch were determined according to Yoshida *et al.* (1976). Approximately 100 mg sample was extracted with 80 % (v/v) aqueous ethanol at 80 ℃ for 30 min. The extract was centrifugated and the supernatant was transferred to a 100 mL volumetric flask. The extraction process was repeated three times; all the three supernatants were pooled into the flask, followed by addition of distilled water to 100 mL. Aliquot of the extract was used for determination of soluble sugars with anthrone reagent. For the starch determination, the residue after centrifugation in the tube was added 2 mL distilled water, and put in a boiling water bath for 15 min. Two millilitres of 9.2 mol L^-1^ HClO_4_ was added to the tube and put into ice bath for 15 min for complete digestion of starch into glucose. Supernatant of the extract was collected into a 100 mL volumetric flask after centrifugation. The extraction was repeated by putting the residue in 2 mL of 4.6 mol L^-1^ HClO_4_ for 15 min for a second time. The supernatants were pooled together in the flask, and then added distilled water to 100 mL. For the colorimetric assay, optical density was measured at 620 nm on a microplate reader (Nano Quant, infinite M200, Tecan, Switzerland). Glucose released in the extraction was estimated with anthrone reagent and converted to starch value by multiplying by 0.9 (Pucher *et al.* 1932). The concentrations of soluble sugars and starch expressed as mg glucose g^-1^ dry weight were calculated by comparing with glucose standard. The non-structural carbohydrates (NSC) concentration of a given plant part refers to the sum of the concentrations of soluble sugars and starch (mg glucose g^-1^ dry weight).

Leaf cell wall fractionation procedure was described by Peng *et al.* (2000) with minor modification (Wu *et al.* 2013). The soluble sugar, lipids and starch of the samples were successively removed with the potassium phosphate buffer (pH = 7.0), chloroform–methanol (1:1, v/v), dimethyl sulphoxide (DMSO)–water (9:1, v/v). The remaining pellet was extracted with 0.5 % (v/v) Oxalic acid for 1 h at 100 °C and washed with 0.5 % (v/v) Oxalic acid and distilled water, all extracts were collected to do colorimetric analysis for determining pectic substance. The remaining pellet was extracted with 4 M KOH containing 1.0 mg/mL sodium borohydride for 1 h at 25 °C and washed with distilled water until the soluble sugars were undetectable. The combined supernatant was neutralized, dialysed and lyophilized as KOH-extractable hemicelluloses. The remaining residues were then extracted with 2 M trifluoroacetic acid at 120 °C in an autoclave for 1 h, and were washed by distilled water. The combined supernatants were collected as the non-KOH-extractable hemicelluloses, and they were combined with the KOH-extractable as total hemicelluloses. The remaining residues were sequentially extracted with acetic acid–nitric acids–water (8:1:2, v/v/v) for 1 h in a boiling water bath and the remaining pellet was defined as cellulose. All samples were conducted in biological triplicate. A UV/VIS Spectrometer (V-1100D, MAPADA Instruments Co., Ltd, Shanghai, China) was applied for total hexoses and pentoses assay as described by Wu *et al.* (2013) and Li *et al.* (2014). The anthrone/H_2_SO_4_ method was used for determination of total hexoses (Fry 1988), and the orcinol/HCl assay was for total pentoses (Dische 1962). The standard curves for hexoses and pentoses were drawn using D-glucose and D-xylose as standard, respectively. Both anthrone/H_2_SO_4_ and orcinol/HCl methods were used to measure total hemicelluloses levels and were also employed for total sugars released from pretreatment and enzymatic hydrolysis of biomass samples. Regarding the high pentoses level effect on the absorbance reading at 620 nm for hexoses account, the deduction from pentoses reading at 660 nm was conducted for final calculation of hexoses level, verified by GC–MS analysis. All of the samples resulted from biological triplicates.

Total lignin contents of the raw samples and the residues obtained from pretreatment were determined by two-step acid hydrolysis method according to Laboratory Analytical Procedure of the National Renewable Energy Laboratory (NREL; Sluiter *et al.* 2008). The acid-insoluble lignin was accounted gravimetrically after correction for ash. The acid-soluble lignin was measured by UV spectroscopy. The details of the two-type of lignin assay were previously described by Xu *et al.* (2012). All samples were performed in biological triplicate.

### Statistical analysis

One-way analysis of variance (ANOVA) was used to assess varietal differences in each parameter using Statistix 9 software (Analytical Software, Tallahassee, FL, USA). Linear regression analysis was performed to test the correlations between parameters using SigmaPlot 10 (Systat Software Inc., CA, USA).

## Results

### Varietal differences of leaf structural traits and their impacts on mesophyll CO_2_ diffusion conductance in rice plants

Leaf structural traits showed significant varietal differences among eight rice genotypes (Table 1 and Fig. 1). *A*_area_, *A*_mass_, PNUE, LMA, LT, LD, *S*_m_, *S*_c_, CWT and *f*_ias_ all showed significant varietal differences, while *g*_m_ showed no varietal difference (Table 1).

**Table 1.** Leaf net photosynthetic rate, photosynthetic nitrogen use efficiency, mesophyll CO_2_ diffusion conductance and anatomical traits of the eight rice genotypes. *A*_area_, area-based net photosynthetic rate; *A*_mass_, mass-based net photosynthetic rate; PNUE, photosynthetic nitrogen use efficiency; g_m_, mesophyll conductance to CO_2_; LMA, leaf mass per area; *S*_m_, the surface area of mesophyll cells exposed to intercellular airspace per leaf area; *S*_c_, the surface area of chloroplasts exposed to intercellular airspaces per leaf area; CWT, cell wall thickness; *f*_ias_, the fraction of intercellular airspaces. *P < 0.05; **P < 0.01; ***P < 0.001.

Genotype	*A*_area_ (μmol m^-2^ s^-1^)	*A*_mass_ (mmol g^-1^ s^-1^)	PNUE (μmol g^-1^ N s^-1^)	*g*_m_ (mol m^-2^ s^-1^)	LMA (g m^-2^)	LT (mm)	LD (mg mm^-3^)	*S*_m_ (μm^2^ μm^-2^)	*S*_c_ (μm^2^ μm^-2^)	CWT (nm)	*f*_ias_ (%)
Sab Ini	28.0 ± 3.9	528 ± 74	15.3 ± 2.1	0.21 ± 0.04	53.0 ± 3.1	0.19 ± 0.01	0.28 ± 0.01	9.4 ± 0.6	8.6 ± 0.5	285 ± 43	18.2 + 4.7
Nucleoryza	28.1 ± 0.7	572 ± 15	18.4 ± 0.5	0.31 ± 0.05	49.2 ± 2.1	0.19 ± 0.01	0.25 ± 0.01	10.0 ± 1.3	9.8 ± 1.2	198 ± 14	24.8 + 1.8
Champa	25.7 ± 2.9	572 ± 65	20.1 ± 2.3	0.27 ± 0.02	45.0 ± 1.1	0.20 ± 0.01	0.23 ± 0.01	10.5 ± 1.5	9.2 ± 1.3	243 ± 26	17.3 + 1.7
Kirmizi Celtik	18.1 ± 0.9	362 ± 19	12.7 ± 0.7	0.27 ± 0.04	49.8 ± 2.1	0.20 ± 0.00	0.25 ± 0.00	9.7 ± 1.1	9.1 ± 1.0	231 ± 29	15.1 + 0.5
Huayou 675	29.8 ± 1.1	611 ± 22	20.1 ± 0.7	0.34 ± 0.01	48.8 ± 3.0	0.17 ± 0.02	0.29 ± 0.04	8.6 ± 1.7	7.8 ± 1.6	287 ± 26	17.7 + 2.3
Huanghuazhan	29.3 ± 3.8	512 ± 67	19.1 ± 2.5	0.23 ± 0.02	57.3 ± 0.3	0.35 ± 0.03	0.17 ± 0.02	19.2 ± 2.5	18.8 ± 2.5	262 ± 20	18.3 + 3.0
Teqing	30.8 ± 2.6	656 ± 56	21.9 ± 1.9	0.28 ± 0.05	46.9 ± 0.9	0.29 ± 0.02	0.16 ± 0.01	14.6 ± 3.8	14.1 ± 3.6	205 ± 21	18.8 + 1.5
Yongyou 12	22.3 ± 2.4	311 ± 33	15.3 ± 1.6	0.18 ± 0.04	71.7 ± 1.1	0.33 ± 0.02	0.21 ± 0.01	19.3 ± 1.6	18.7 ± 1.5	330 ± 22	20.3 + 1.5
ANOVA											
Average	26.5	516	17.9	0.26	52.7	0.24	0.23	12.7	12.0	257	18.8
Genotype	***	***	***	ns	***	***	***	***	***	***	**

**Fig. 2.**
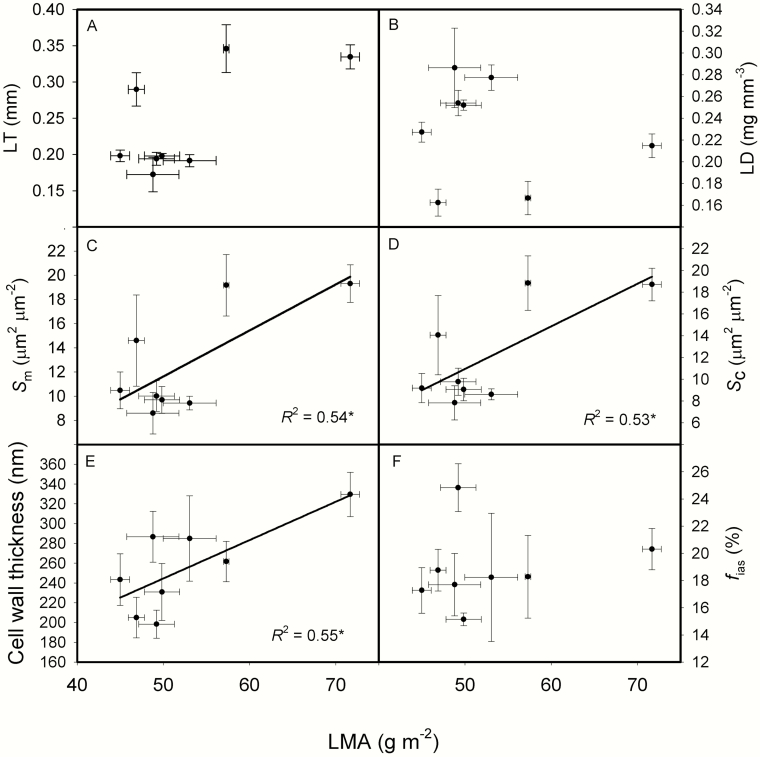
The relationships between leaf mass per area (LMA) and leaf thickness (LT, A), leaf density (LD, B), the surface area of mesophyll cells exposed to intercellular airspaces per leaf area (*S*_m_, C), the surface area of chloroplasts exposed to intercellular airspaces per leaf area (*S*_c_, D), cell wall thickness (CWT, E) and the fraction of intercellular airspaces (*f*_ias_, F) across the eight rice genotypes. Data are means ± SD of three replicates for LMA, LT, LD, *S*_m_, *S*_c_ and *f*_ias_. CWT of each genotype was measured with 5–13 pictures, and one mesophyll cell was measured in each image. *P < 0.05; **P < 0.01; ***P < 0.001.

**Fig. 3.**
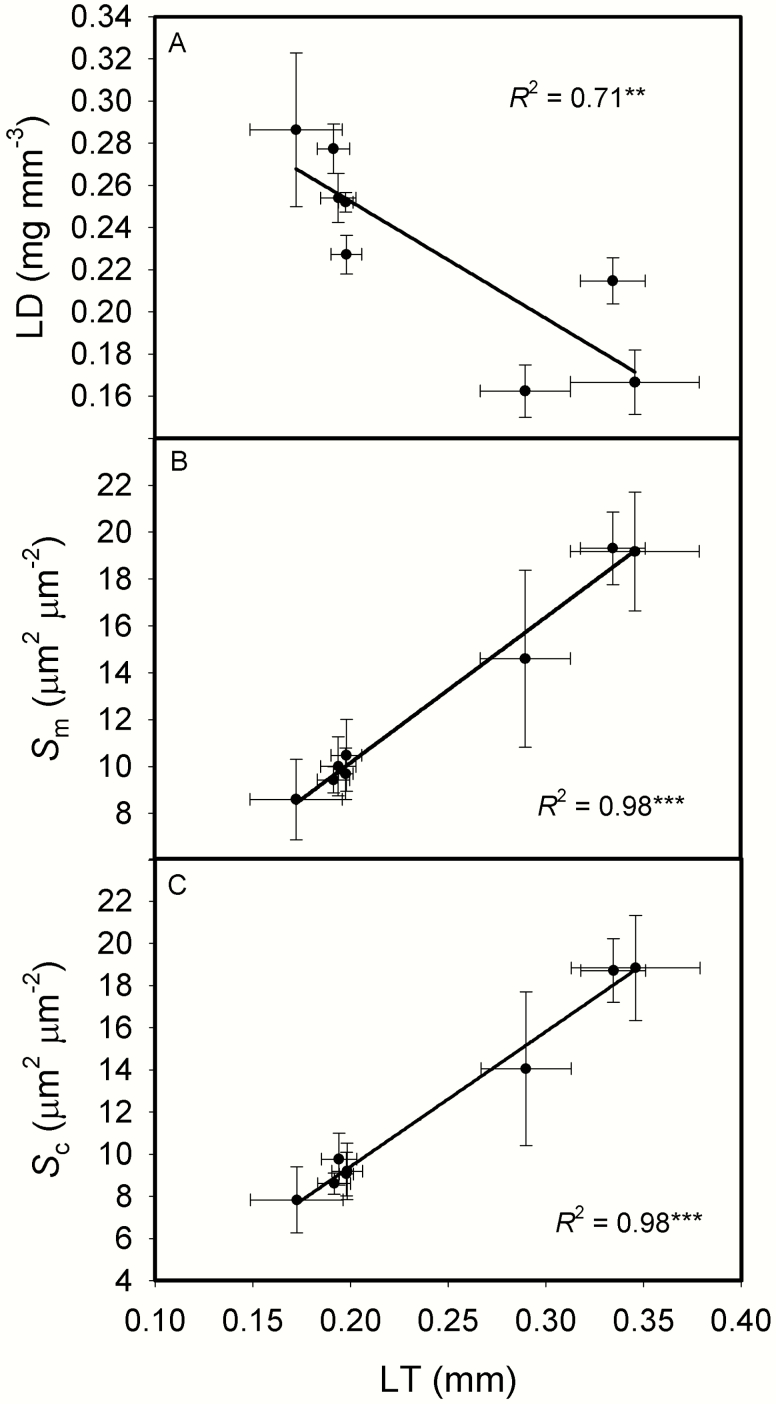
The relationships between leaf thickness (LT) and leaf density (LD, A), the surface area of mesophyll cells exposed to intercellular airspaces per leaf area (*S*_m_, B), the surface area of chloroplasts exposed to intercellular airspaces per leaf area (*S*_c_, C) across the eight rice genotypes. Data are means ± SD of three replicates. *P < 0.05; **P < 0.01; ***P < 0.001.

For the relationships between leaf structure and anatomical traits, LT was marginally correlated with LMA (Fig. 2A, *P* = 0.0688). *S*_m_, *S*_c_ and CWT all increased as LMA increased (Fig. 2C–E). As LT increased, LD decreased, while *S*_m_ and *S*_c_ significantly increased (Fig. 3).

**Fig. 4.**
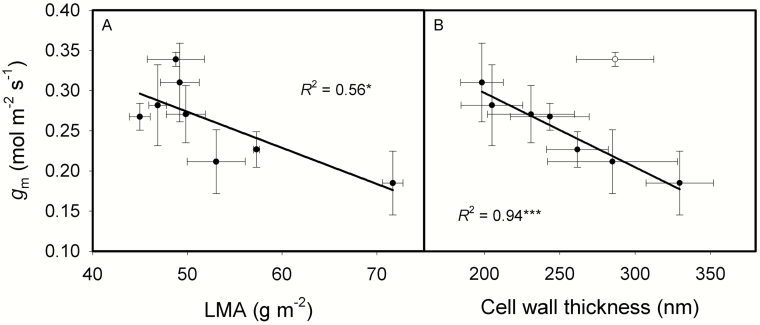
The relationships between mesophyll conductance (*g*_m_) and leaf mass per area (LMA, A), cell wall thickness (B) across the eight rice genotypes. Data are means ± SD of three replicates for gm and LMA. Cell wall thickness of each genotype was measured with 5-13 pictures, and one mesophyll cell was measured in each picture. *P < 0.05; **P < 0.01; ***P < 0.001.

**Fig. 5.**
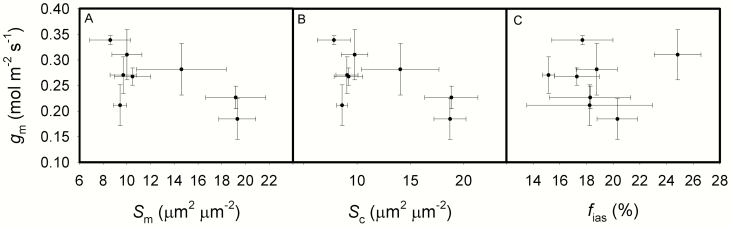
The relationships between mesophyll conductance (*g*_m_) and the mesophyll cells surface area exposed to intercellular airspaces per leaf area (*S*_m_, A), the surface area of chloroplasts exposed to intercellular airspaces per leaf area (*S*_c_, B), the fraction of intercellular airspaces (*f*_ias_, C) across the eight rice genotypes. Data are means ± SD of three replicates. *P < 0.05; **P < 0.01; ***P < 0.001.

For the impacts of leaf structure and anatomy on CO_2_ diffusion conductance, *g*_m_ decreased as LMA increased (Fig. 4A). *g*_m_ was not correlated with CWT (*P* = 0.1453), when excluding the outlier of Huayou 675, however, *g*_m_ was negatively correlated with CWT (Fig. 4B). *S*_m_, *S*_c_ and *f*_ias_ showed no effects on *g*_m_ (Fig. 5) and *A*_area_ (data not show).

### Leaf chemical compositions in eight rice genotypes

Leaf chemical compositions including pectic substance, hemicellulose, cellulose, NSC (soluble sugar and starch) showed significant varietal differences on both a leaf-area and mass basis, except for lignin which was only significant on a mass basis [**see **Supporting Information—Tables S1**and**S2].

### The tradeoff between structural tissues and intracellular chemical components as well as its impact on leaf photosynthetic rate

**Fig. 6.**
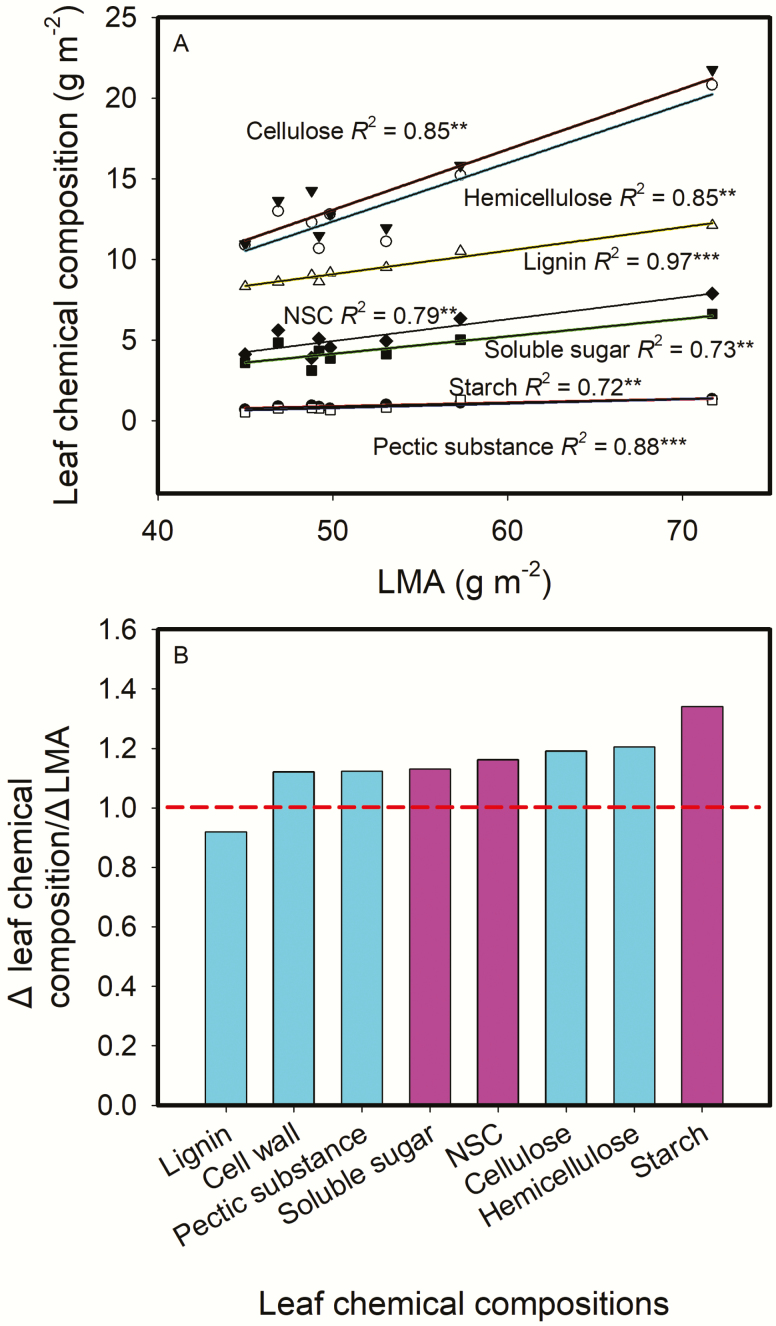
Relationships between leaf chemical compositions (based on leaf area) and leaf mass per area (LMA) across the eight rice genotypes (A), and the ratio of Δleaf chemical composition to ΔLMA (B). ΔLMA was calculated as the ratio of the maximum to the minimum LMA across the tested rice genotypes. The obtained regression equations in (A) were used to calculate leaf chemical compositions with the maximum and the minimum LMA, respectively, and Δ leaf chemical composition was calculated as the ratio of the maximum to the minimum leaf chemical compositions. Data are means of three replicates in (A). *P < 0.05; **P < 0.01; ***P < 0.001.

**Fig. 7.**
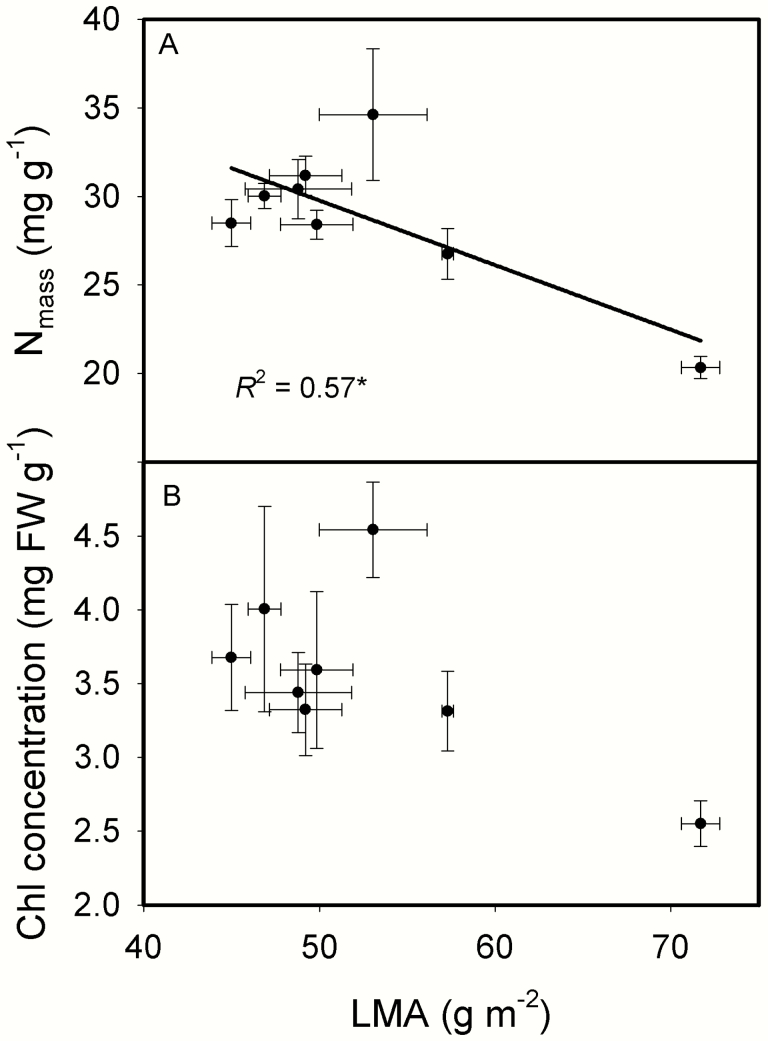
The relationships between leaf mass per area (LMA) and leaf N content based on leaf mass (N_mass_, A), leaf chlorophyll content based on leaf mass (B) across the eight rice genotypes. Data are means ± SD of three replicates. *P < 0.05; **P < 0.01; ***P < 0.001.

**Fig. 8.**
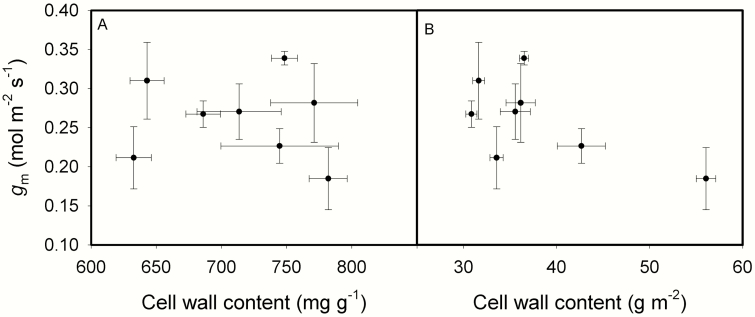
The relationships between mesophyll conductance (*g*_m_) and mass-based cell wall content (A) and area-based cell wall content (B) across the eight rice genotypes. Data are means ± SD of three replicates.

**Fig. 9.**
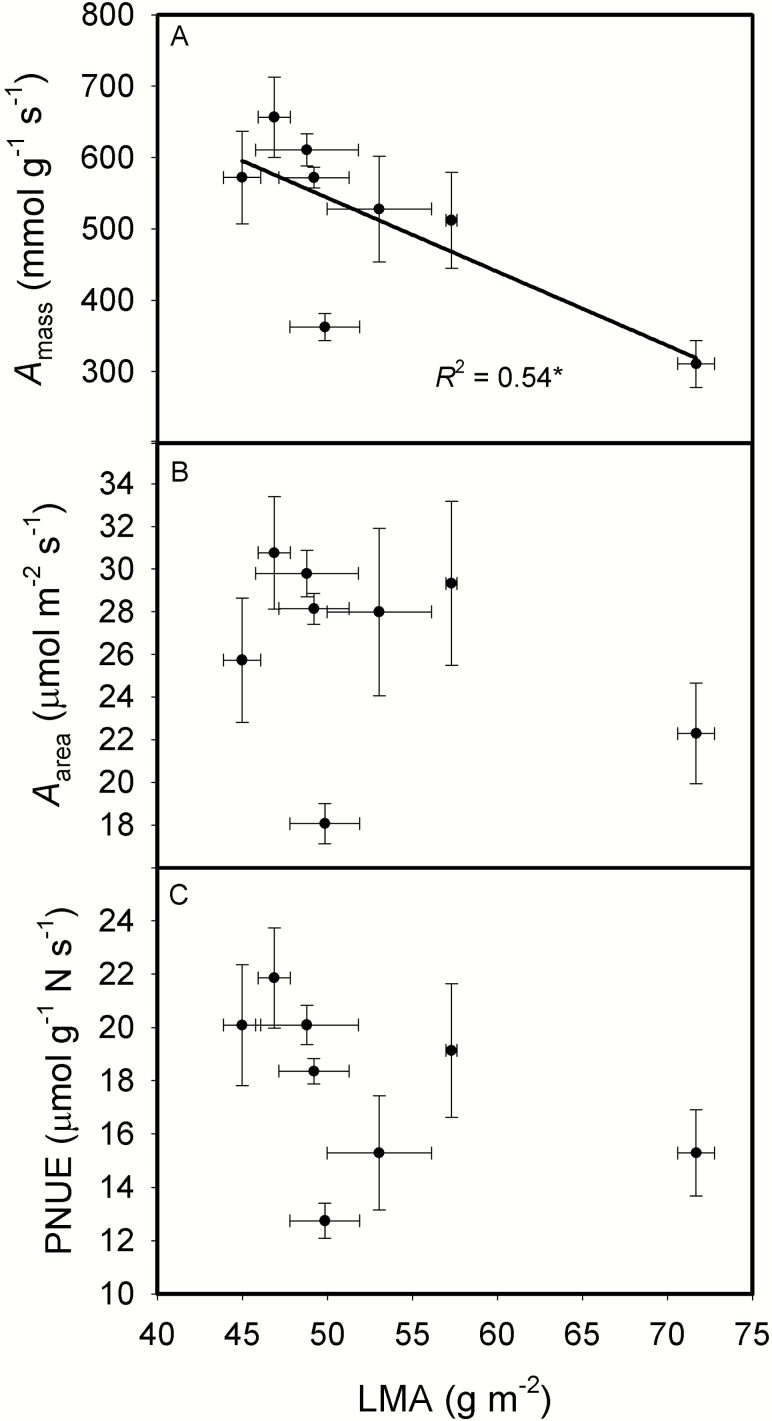
The relationships between leaf mass per area (LMA) and mass-based net photosynthetic rate (*A*_mass_, A), area-based net photosynthetic rate (*A*_area_, B) and photosynthetic nitrogen use efficiency (PNUE, C) across the eight rice genotypes. Data are means ± SD of three replicates. *P < 0.05; **P < 0.01; ***P < 0.001.

Figure 6A showed that the contents of NSC, and leaf cell wall compounds including pectic substance, hemicellulose, cellulose and lignin expressed on a leaf-area all increased as LMA increased. The relative increasing ratios of leaf chemical compositions to LMA were shown in Fig. 6B. Except for lignin, the relative increasing ratios of other three cell wall compounds and NSC to LMA were all above one, meaning that the changes in these chemical compositions were larger than the change in LMA, the concentrations of most cell wall compounds and NCS based on leaf mass increased as LMA increased. On the other hand, leaf N content based on leaf mass (N_mass_) decreased as LMA increased, and leaf chlorophyll content based on leaf mass (mg FW g^-1^) showed a downtrend as LMA increased (Fig. 7). *g*_m_ was not correlated with either mass-based or area-based cell wall content (Fig. 8). *A*_mass_ decreased as LMA increased, while *A*_area_ and PNUE showed no relationships with LMA (Fig. 9). In addition, *A*_area_ showed positive relationship with stomatal conductance to H_2_O (*g*_s_), but no relationships with N_area_ and *g*_m_**[see **Supporting Information—Fig. S1**]**.

Moreover, it was observed that leaf mesophyll cell wall compounds accounted for more than half of the leaf dry mass, pectic substance accounted for 1.8 %, hemicellulose accounted for 25.1 %, cellulose accounted for 26.5 %, lignin accounted for 18.1 % and total cell wall compounds accounted for 71.5 % **[see **Supporting Information—Fig. S2**]**. In addition, leaf NSC accounted for 10.0 % of leaf dry mass, as soluble sugar accounted for 8.4 % and starch accounted for 1.6 %.

## Discussion

Mesophyll conductance (*g*_m_) is an important CO_2_ diffusion conductance and limits photosynthesis in the same magnitude as stomatal conductance (Evans and von Caemmerer 1996; Evans and Loreto 2000; Flexas *et al.* 2008; Terashima *et al.* 2011). In the last decades, leaf anatomical traits including *S*_m_, *S*_c_, the fraction of intercellular airspace (*f*_ias_) and leaf cell wall thickness were proved to have an impact on *g*_m_ (Evans *et al.* 2009; Scafaro *et al.* 2011; Terashima *et al.* 2011; Peguero-Pina *et al.* 2012; Tosens *et al.* 2012; Muir *et al.* 2014; Ren *et al.* 2019). LMA is an integrated leaf anatomical trait, and the influence of LMA on area-based gas exchange parameters is inconsistent in previous studies (Hikosaka *et al.* 2009; Liu and Li 2016; Onoda *et al.* 2017; Ye *et al.* 2019; Ren *et al.* 2019). Moreover, there is no concensus as to which factor drives the most variation in LMA (Choong *et al.* 1992; Castro-Díez *et al.* 2000; Villar *et al.* 2013). Poorter *et al.* (2010) showed that high LMA species have more structural compositions and less inclusions such as organic acids, minerals and protein. Further, using data of dry mass fraction in cell walls, nitrogen allocation, mesophyll CO_2_ diffusion and associated anatomical traits from hundreds of species, Onoda *et al.* (2017) concluded that high LMA species have more cell wall constituents, and thicker cell wall showed lower *g*_m_.

The variation of LMA in the cultivated rice genotypes is relatively small, although wild rice may potentially show a large variation in LMA (Xiong *et al.* 2016*b*). In the present study, LT increased with LMA across eight rice genotypes, while LD and *f*_ias_ did not respond to LMA. This indicates that in this case the increase in LMA is more closely related to an increase in LT, rather than LD. This is contrary to results in Xiong *et al.* (2016*b*), which showed that LD rather than LT is the main driving factor for the variation in LMA in rice leaves. The inconsistent results may relate to different materials and treatments. In the study by Xiong *et al.* (2016*b*), 11 rice genotypes were supplied with sufficient nitrogen and four were supplied with low nitrogen; moreover, five wild rice genotypes, which possess a broad range of leaf traits values, were also studied. For example, LD showed 4.4-fold difference in Xiong *et al.* (2016*b*), while it varied from 0.17 to 0.29 mg mm^-3^ in the present study.

In the present study, *S*_m_, *S*_c_ and CWT all increased with LMA (Fig. 2). However, these positive relationships were mainly driven by the cultivar of Yongyou 12 (Fig. 2), which possessed different anatomical traits. More genotypes should be included in future to support these relationships in rice. It was reported that cell wall resistance is responsible for about half of the total mesophyll resistance (Terashima *et al.* 2011). Cell wall thickness ranged from 198 to 330 nm in the present study (Table 1), and *g*_m_ was significantly and negatively correlated to CWT (Fig. 4). This suggested that the correlation between *g*_m_ and LMA is more driven by the CWT (Niinemets *et al.* 2009; Veromann-Jurgenson *et al*. 2017) in comparison with *S*_m_ and *S*_c_. In addition to CWT, cell wall porosity may have an important role in regulating *g*_m_ (Ellsworth *et al.* 2018), although cell wall porosity cannot be directly measured and its variation among different genotypes is not known. *g*_m_ of Huayou 675 can reach as high as 0.34 mol m^-2^ s^-1^, although the CWT was 287 nm. The reason for Huayou 675 to possess a high *g*_m_ with a thick cell wall is unknown, this cultivar may possess a high cell wall porosity in comparison with other genotypes or a relative higher aquaporin function. The genotype of Yongyou 12 had a thick cell wall, a low *g*_m_ and a low *A*_mass_ (Table 1). Even though Yongyou 12 cannot reach a high photosynthetic rate, it does not imply that it cannot reach a high yield. Thick cell wall can improve the toughness of leaves, the tolerance to physical disturbance, can protect plants from herbivores and pathogens and finally leads to a long life span (Coley 1983; Reich *et al.* 1991; Wright and Cannon 2001; Onoda *et al*. 2008, 2017; Hikosaka *et al.* 2009). It was reported that photosynthesis in rice plants during the grain-filling period contributes 60–100 % of the final grain carbon content (Yoshida 1981). So, if the leaves can keep photosynthesis for a longer duration, it can produce more rice grain yield. Therefore, though Yongyou 12 has thick cell wall (330 nm) and a relative low photosynthetic rate (22.3 μmol m^-2^ s^-1^), it still shows high grain yield in previous studies (Wang *et al.* 2014; Wei *et al.* 2014).

For leaf chemical compositions, they can be expressed as mass-based content or area-based content. The mass-based compositions content should keep constant, if the increments of the chemical compositions are proportional to the increment of LMA. However, it is seldom the case (Poorter *et al.* 2010). In the present study, the concentrations of most cell wall compounds based on leaf mass did not change with LMA, but the relative increasing ratios of leaf chemical compositions to LMA were above 1 for cell wall compounds (except for lignin) and NSC, while Fig. 7 showed that leaf nitrogen and leaf chlorophyll contents based on mass decreased with LMA. This suggested that rice genotypes with a high LMA would possess more structural material and less nitrogen and chlorophyll. These results were consistent with the previous studies showing that high LMA leaves tended to have more cell wall components (Mommer *et al.* 2005; Mediavilla *et al.* 2008). However, *g*_m_ was not correlated with area-based cell wall content in the present study (Fig. 8). Not only determined by leaf anatomical traits, *g*_m_ is also affected by biochemical components such as aquaporins, carbonic anhydrase, etc. (Nakhoul *et al.* 1998; Uehlein 2003; Evans *et al.* 2009). Thus, the non-significant correlation between *g*_m_ and cell wall content may be caused by the compensating effects of aquaporins or other biochemical components yet to be investigated.

Moreover, LD decreased as LT increased (Fig. 3A). *g*_m_ and photosynthetic rate would be low with thick cell walls, low leaf nitrogen and chlorophyll concentrations. As shown in Fig. 4, *g*_m_ significantly decreased with increasing LMA and cell wall thickness. LMA has no effect on *A*_area_ and PNUE, though *A*_mass_ decreased with LMA (Fig. 9), these results are in agreement with previous studies (Wright *et al.* 2004; Ren *et al.* 2019). The reason why *g*_m_ was not correlated with *A*_area_ may be that *g*_s_ was the main determinant for *A*_area_ in the present study **[see **Supporting Information—Fig. S1**]**.

For the percentages of different leaf chemical compositions to leaf dry mass, cell wall compounds accounted for 71.5 % of leaf dry mass, NSC accounted for 10.0 % of leaf dry mass in the present study. This result was consistent with the study of Onoda *et al.* (2017), which illustrated that cell wall constituents are major components of leaf dry mass (18–70 %). LMA not only has an effect on the fraction of dry mass between structural tissues and inclusions, it can also have an effect on nitrogen allocation and photosynthesis. Onoda *et al.* (2017) reported that high fraction of leaf mass in cell wall is typically associated with a lower fraction of leaf N invested in photosynthetic proteins, and lower within-leaf CO_2_ diffusion rates. Takashima *et al.* (2004) also showed that species with a longer leaf life span have a greater LMA, lower photosynthetic capacity and lower PNUE. Nitrogen allocation into each chemical composition have not been estimated in the present study and should be explored in future for rice to assess the impacts of nitrogen allocation on PNUE, among others.

## Conclusions

This study reports evidence that high LMA rice plants invest more leaf mass to cell wall and possess a low mesophyll conductance. There were significant intraspecific variations of leaf anatomy in rice plants. LT but not LD was the main driving factor for different LMA in the present study. *S*_m_, *S*_c_ and cell wall thickness all increased as LT increased. Thick cell wall as a result of more mass was investing to cell wall but less to leaf nitrogen and chlorophyll has led to lower *g*_m_ and *A*_mass_. Cell wall compounds accounted for most of the leaf dry mass in rice leaves. The mechanism of high *g*_m_ with thick cell wall (like Huayou 675) should be explored in future.

## Supporting Information

The following supporting information is available in the online version of this article—

**Figure S1**. The relationships between area-based leaf photosynthetic rate (*A*_area_) and area- based leaf nitrogen content (N_area_, A), stomatal conductance to H_2_O (*g*_s_, B) and mesophyll conductance (*g*_m_, C) across the eight rice genotypes.

**Figure S2**. Percentages of leaf chemical compositions to leaf dry mass in the eight rice genotypes.

**Table S1**. Leaf chemical composition per unit leaf mass in eight rice genotypes.

**Table S2**. Leaf chemical composition per unit leaf area in eight rice genotypes.

plaa028_suppl_Supplementary_Material

## Notes

Form & Function. Chief Editor: Kate McCulloh
