# Supplementary material for: High leaf mass per area Oryza genotypes invest more leaf mass to cell wall and show a low mesophyll conductance
Source: AoB Plants. 2020 Jun 19;12(4):plaa028. doi: 10.1093/aobpla/plaa028 (PMC7396964; doi:10.1093/aobpla/plaa028)
Supplement: plaa028_suppl_Supplementary_Material [file plaa028_suppl_supplementary_material.pdf]

Supplementary data

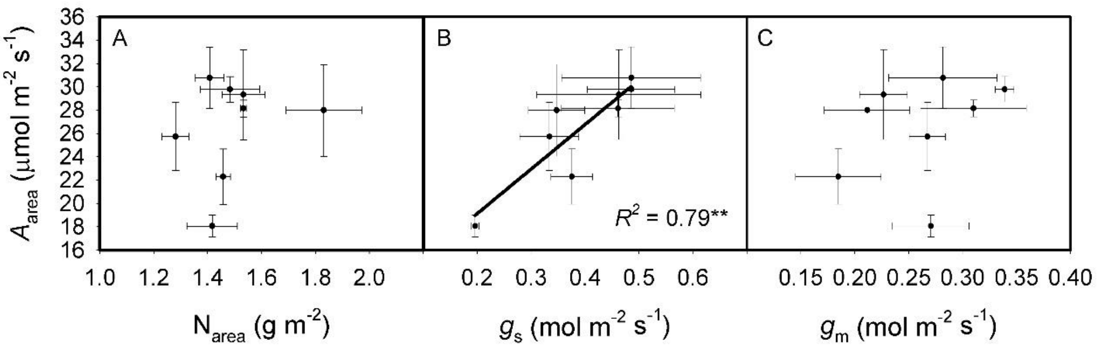

Fig. S1. The relationships between area-based leaf photosynthetic rate (A_{area}) and area-based leaf nitrogen content (N_{area}, A), stomatal conductance (g_s, B) and mesophyll conductance (g_m, C) across the 8 rice genotypes. Data are means ± SD of 3 replicates. *, $P < 0.05$; **, $P < 0.01$; ***, $P < 0.001$.

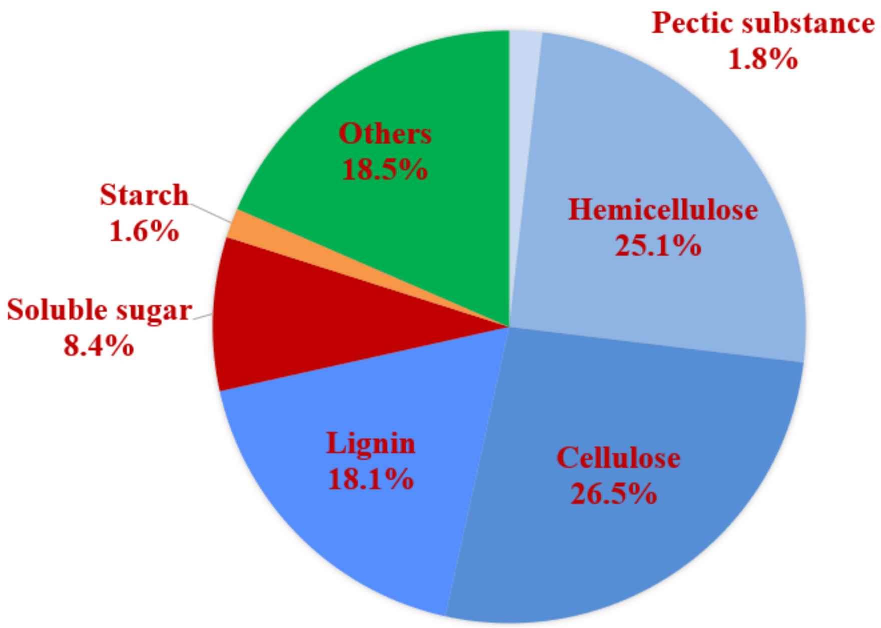

Fig. S2. Percentages of leaf chemical compositions to leaf dry mass in the 8 rice genotypes.

Table S1 Leaf chemical composition per unit leaf mass in eight rice genotypes.

Genotype	Pectic substance ($mg\ g^{-1}$)	Hemicellulose ($mg\ g^{-1}$)	Cellulose ($mg\ g^{-1}$)	Lignin ($mg\ g^{-1}$)	Cell wall ($mg\ g^{-1}$)	Soluble sugar ($mg\ g^{-1}$)	Starch ($mg\ g^{-1}$)	NSC ($mg\ g^{-1}$)	Nitrogen ($mg\ g^{-1}$)	Chlorophyll （$mg\ g^{-1}$ FW)
Sab Ini	18.9 ± 1.7	209 ± 9.0	225.4 ± 10.5	179.1 ± 5.7	632.6 ± 13.5	77.8 ± 8.3	15.6 ±2.9	93.5 ± 5.6	34.6 ± 3.7	4.5 ± 0.3
Nucleoryza	17.3 ± 0.5	217.1 ± 8.9	233.4 ± 11.9	175.1 ± 6.8	642.9 ± 13.1	88.1 ± 6.5	15.5 ±1.6	103.6 ± 5.5	31.2 ± 1.1	3.3 ± 0.3
Champa	15.4 ± 1.7	242.1 ± 10.0	243.4 ± 3.3	185.0 ± 3.4	685.9 ± 13.2	80.1 ± 5.5	11.7 ±1.7	91.8 ± 6.0	28.5 ± 1.3	3.7 ± 0.4
Kirmizi Celtik	15.6 ± 3.8	256.8 ± 13.8	257.1 ± 12.3	184.1 ± 13.2	713.6 ± 32.4	77.8 ± 4.0	13.3 ±2.1	91.1 ± 2.1	28.4 ± 0.8	3.6 ± 0.5
Huayou 675	19.3 ± 0.3	251.8 ± 5.3	292.2 ± 4.2	185.1 ± 14.3	748.4 ± 10.0	63.8 ± 3.8	16.1 ±0.6	79.9 ± 4.3	30.4 ± 1.7	3.4 ± 0.3
Huanghuazhan	19.4 ± 0.2	265.5 ± 2.3	276.3 ± 27.8	183.4 ± 28.0	744.6 ± 45.1	87.8 ± 10.2	22.8 ±2.1	110.6 ± 12.3	26.8 ± 1.4	3.3 ± 0.3
Teqing	19.0 ± 0.5	277.0 ± 3.0	291.5 ± 14.5	183.6 ± 19.7	771.1 ± 33.4	103.4 ± 20.3	16.2 ±3.7	119.6 ± 19.5	30.0 ± 0.7	4.0 ± 0.7
Yongyou 12	18.9 ± 0.8	290.3 ± 3.2	303.7 ± 12.1	169.0 ± 0.2	781.9 ± 14.6	92.4 ± 11.0	17.7 ±4.1	110.1 ± 10.3	20.3 ± 0.6	2.6 ± 0.2
ANOVA										
Average	18.0	251.2	265.4	180.6	715.1	83.9	16.1	100.0	28.8	3.6
Genotype	*	***	***	ns	***	*	*	**	***	**

The content of leaf chlorophyll is based on leaf fresh weight, others are based on dry weight. Cell wall compounds include pectic substance, hemicellulose, cellulose and lignin. NSC, Non-structural carbohydrate, includes soluble sugar and Starch. *, $P < 0.05$; **, $P < 0.01$; ***, $P < 0.001$.

Table S2 Leaf chemical composition per unit leaf area in eight rice genotypes.

Genotype	Pectic substance (g m^{-2})	Hemicellulose (g m^{-2})	Cellulose (g m^{-2})	Lignin (g m^{-2})	Cell wall (g m^{-2})	Soluble sugar (g m^{-2})	Starch (g m^{-2})	NSC (g m^{-2})	Nitrogen (g m^{-2})	Chlorophyll (mg m^{-2})
Sab Ini	1.0 ± 0.1	11.1 ± 0.5	12.0 ± 0.6	9.5 ± 0.3	33.6 ± 0.7	4.1 ± 0.4	0.8 ± 0.2	5.0 ± 0.3	1.83 ± 0.14	683 ± 49
Nucleoryza	0.9 ± 0.0	10.7 ± 0.4	11.5 ± 0.6	8.6 ± 0.3	31.6 ± 0.6	4.3 ± 0.3	0.8 ± 0.1	5.1 ± 0.3	1.53 ± 0.01	407 ± 38
Champa	0.7 ± 0.1	10.9 ± 0.4	10.9 ± 0.1	8.3 ± 0.2	30.9 ± 0.6	3.6 ± 0.2	0.5 ± 0.1	4.1 ± 0.3	1.28 ± 0.05	473 ± 46
Kirmizi Celtik	0.8 ± 0.2	12.8 ± 0.7	12.8 ± 0.6	9.2 ± 0.7	35.6 ± 1.6	3.9 ± 0.2	0.7 ± 0.1	4.5 ± 0.1	1.42 ± 0.09	518 ± 77
Huayou 675	0.9 ± 0.0	12.3 ± 0.3	14.2 ± 0.2	9.0 ± 0.7	36.5 ± 0.5	3.1 ± 0.2	0.8 ± 0.0	3.9 ± 0.2	1.48 ± 0.11	439 ± 35
Huanghuazhan	1.1 ± 0.0	15.2 ± 0.1	15.8 ± 1.6	10.5 ± 1.6	42.7 ± 2.6	5.0 ± 0.6	1.3 ± 0.1	6.3 ± 0.7	1.53 ± 0.08	459 ± 38
Teqing	0.9 ± 0.0	13.0 ± 0.1	13.7 ± 0.7	8.6 ± 0.9	36.1 ± 1.6	4.8 ± 1.0	0.8 ± 0.2	5.6 ± 0.9	1.41 ± 0.05	498 ± 86
Yongyou 12	1.4 ± 0.1	20.8 ± 0.2	21.8 ± 0.9	12.1 ± 0.0	56.1 ± 1.0	6.6 ± 0.8	1.3 ± 0.3	7.9 ± 0.7	1.46 ± 0.03	445 ± 27
ANOVA										
Average	1.0	13.3	14.1	9.5	37.9	4.4	0.9	5.3	1.49	490
Genotype	***	***	***	***	***	***	***	***	***	***

Cell wall compounds include pectic substance, hemicellulose, cellulose and lignin. NSC, Non-structural carbohydrate, includes soluble sugar and Starch. *, $P < 0.05$; **, $P < 0.01$; ***, $P < 0.001$.
